# Prevalence and Antimicrobial Resistance of Causative Agents to Ocular Infections

**DOI:** 10.3390/antibiotics11040463

**Published:** 2022-03-30

**Authors:** Roberta Manente, Biagio Santella, Pasquale Pagliano, Emanuela Santoro, Vincenzo Casolaro, Anna Borrelli, Mario Capunzo, Massimiliano Galdiero, Gianluigi Franci, Giovanni Boccia

**Affiliations:** 1 Section of Microbiology and Virology, University Hospital “Luigi Vanvitelli”, 80138 Naples, Italy; roberta.manente@studenti.unicampania.it (R.M.); bi.santella@gmail.com (B.S.); massimiliano.galdiero@unicampania.it (M.G.); 2 Department of Medicine, Surgery and Dentistry “Scuola Medica Salernitana”, University of Salerno, 84081 Baronissi, Italy; ppagliano@unisa.it (P.P.); esantoro@unisa.it (E.S.); vcasolaro@unisa.it (V.C.); mcapunzo@unisa.it (M.C.); 3 Azienda Ospedaliero Universitaria San Giovanni di Dio e Ruggi D’Aragona, 84131 Salerno, Italy; anna.borrelli@sangiovannieruggi.it; 4 Dai Dipartimento Di Igiene Sanitaria e Medicina Valutativa U.O.C. Patologia Clinica e Microbiologica, Azienda Ospedaliero-Universitaria S. Giovanni di Dio e Ruggi D’Aragona Scuola Medica Salernitana, Largo Città di Ippocrate, 84131 Salerno, Italy; 5 Department of Experimental Medicine, University of Campania “Luigi Vanvitelli”, 80138 Naples, Italy

**Keywords:** ocular infection, bacteria, antibiotics, antimicrobial stewardship, cross-sectional study

## Abstract

Bacterial ocular infections are a worldwide health problem and, if untreated, can damage the structure of the eye and contribute to permanent disability. Knowledge of the prevalence and antimicrobial susceptibility patterns of the main causative agents involved in ocular infections is necessary for defining an optimal antibiotic therapy. The aim of this study was to analyse bacterial species involved in ocular infections and the antimicrobial susceptibility patterns. Conjunctival swab samples were collected from patients with bacterial conjunctivitis at the University Hospital San Giovanni di Dio e Ruggi d’Aragona between January 2015 and December 2019. The identification and antibiotic sensitivity tests were performed using the VITEK 2 system. A total of 281 causative agents of ocular infections were isolated, 81.8% of which were Gram-positive bacteria. Coagulase-negative staphylococci (CoNS) were the most commonly isolated species among Gram-positive bacteria, followed by *Staphylococcus aureus.* In contrast, *Pseudomonas* spp. and *Escherichia coli* were the main species isolated among Gram-negative bacteria (18.2%). Overall, linezolid, teicoplanin, tigecycline and vancomycin were the most effective antimicrobials. Analysis of resistance rates over time highlighted increasing resistance for azithromycin, clarithromycin and erythromycin among CoNS, and clindamycin and erythromycin among *Staphylococcus aureus.* This study has identified the profiles of the major pathogens involved in ocular infection and their susceptibility patterns, which will help improve the treatments and the choice of antibiotics in ocular infections.

## 1. Introduction

Ocular infections can damage the anatomic structure of the eye at multiple levels. They are a worldwide health problem, with approximately six million people suffering from blindness or moderate/severe visual impairment [1]. Conjunctivitis is the most frequent ocular infection with noticeable economic and social impact, following keratitis, exogenous endophthalmitis, blepharitis and dacryocystitis [2,3,4,5]. *Staphylococcus aureus* (*S. aureus*), Coagulase-negative staphylococci (CoNS), *Streptococcus pneumoniae* (*S. pneumoniae*) and *Haemophilus influenzae* (*H. influenzae*) are common causative agents of conjunctivitis [6]. In contrast, *Pseudomonas aeruginosa* (*P. aeruginosa*) is the main cause of microbial keratitis [7]. CoNS have been isolated with the highest frequency in polymicrobial infections [8]. *Corynebacterium* and *Propionibacterium acnes* are the species most commonly associated with blepharitis; *S. aureus* and *Streptococcus viridans* are the most common causes of endophthalmitis [9,10]. CoNS and *S. aureus* are associated with all types of eye infections, and several studies have highlighted that they are the main causative agents of these infections [3,11,12]. The most frequently used antibiotic classes to treat ocular infections are β-lactam, aminoglycosides, fluoroquinolones, sulfonamides and tetracyclines, but in recent years, there has been a noticeable increase in resistance rates to these antibiotics [13,14]. A rapid increase in methicillin resistance rates in *S. aureus* and CoNS isolates was reported in recent articles [15,16]. These resistances lead to the failure of first-line antibiotics, with serious complications, such as corneal perforations, endophthalmitis and flap fusion after refractive surgery [17]. Many surveillance studies, such as Antimicrobial Surveillance Program (SENTRY), Study for Monitoring Antimicrobial Resistance Trend (SMART), Tracking Resistance in the United States Today (TRUST) and Antibiotic Resistance Monitoring in Ocular Microorganisms (ARMOR), have been conducted to investigate the increased resistance to antibiotics [18]. In particular, the ARMOR surveillance study evaluated the antibiotic resistance profiles of bacterial isolates from eye infections from 2009 to 2016. A small but significant decrease in resistance rates among Gram-negative bacteria and an increasing rate of resistance to oxacillin and azithromycin among Gram-positive bacteria were observed [6,15]. Finally, antibiotic resistance remains high among conjunctival isolates, particularly among *S. aureus* and CoNS pathogens [19]. The aims of this study were to identify the prevalence and antimicrobial susceptibility patterns of the main causative agents of ocular infections and to define an optimal antibiotic therapy.

## 2. Results

Out of 1364 conjunctival swabs, bacterial growth was observed in 285 samples (21%) (Table 1).

Positive samples from males represented 54.7% of the total samples. About 46% were from patients aged 61–90 years (Table 2).

Among the bacteria isolates, 81.1% were Gram-positive. Among these, CoNS (*Staphylococcus epidermidis, Staphylococcus haemolyticus and Staphylococcus hominis*) were the main species isolated, followed by *S. aureus* (33%). In contrast, *Pseudomonas* spp. (26%) and *Escherichia coli* (14%) were the major species isolated among Gram-negative bacteria (Table 3).

The antimicrobial resistance patterns of *S. aureus* and CoNS are shown in Table 4 and Table 5. The resistance to oxacillin shows rates ranging from 13% to 40%. The rates of resistance to azithromycin, clarithromycin, clindamycin and erythromycin ranged from 41.2 to 50%, from 41.2 to 50%, from 44.4 to 50% and from 44.4 to 50%, respectively. Gentamicin and levofloxacin resistance rates were fluctuating but lower, at 37%. Resistance to vancomycin and resistance to linezolid, rifampicin, tigecycline, and trimethoprim/sulfamethoxazole were not observed.

Among CoNS, the highest resistance rates to azithromycin, clarithromycin and erythromycin were observed in 2018. Moreover, fluctuations in resistance rates to fusidic acid (36.4 to 16.7%), clindamycin (45.5 to 8.3%), levofloxacin (45.5 to 33.3%), oxacillin (57.6 to 41.7%) and tetracycline (3.4 to 16.7%) have been found. The resistance rate to vancomycin was 3% in 2015 and was not found in any other cases the following years. No resistance was found to daptomycin, linezolid or tigecycline. Gram-negative isolates showed low rates of resistance to common antibiotics tested, except for amoxicillin/clavulanic acid, colistin and fosfomycin. Rather, the increase in the rates of resistance to colistin (13.3 to 20.0%) and fosfomycin (9.1 to 25.0%) should be highlighted (Table 6).

## 3. Discussion

Bacteria contribute to 50–70% of eye infections, which, if left untreated, can cause irreversible damage to the eye structure [9,20]. The identification of the responsible bacteria and their antimicrobial susceptibility patterns is essential in establishing an accurate antibiotic therapy for the treatment of ocular infections [21,22]. In this study, 285 bacteria were isolated from ocular swabs, and 81.1% were Gram-positive species. Several studies reported that Gram-positive bacteria were the major species isolated in patients with ocular infections; among them, the staphylococci were the main isolated species. In contrast, Gram-negative bacteria were reported with lower frequency, but *P. aeruginosa* and *E. coli* exhibited high resistance rates and could be isolated in severe cases [3]. Additionally, in this study, Gram-negative bacteria were isolated with a lower frequency and showed low rates of resistance to common antibiotics tested but increased rates of resistance to colistin (13.3 to 20.0%) and fosfomycin (9.1 to 25.5%).

A 15-year review of cases documented in East China indicated that the major pathogens in ocular infections were staphylococci [23]. Similar studies conducted in Iran and India have highlighted that 40% and 45.4% of infections, respectively, were due to CoNS [3]. Other studies performed in low-income settings, such as Ethiopia, indicated *S. aureus* as the predominant isolated pathogen [24]. In our analysis, *S. aureus* showed a higher rate of resistance against penicillin G (84.2%), and rates of resistance to azithromycin, clarithromycin, clindamycin and erythromycin exceeding 40%. All strains were susceptible to linezolid, rifampicin, tigecycline, trimethoprim/sulfamethoxazole and vancomycin. For CoNS, increasing resistance rates to azithromycin, clarithromycin and erythromycin were observed, while no resistance was found to daptomycin, linezolid or tigecycline. 

Furthermore, an important rate of resistance to oxacillin was found (55.5% to 41.7%). Hsu et al. reported that resistance to methicillin and oxacillin was often associated with multidrug resistance [25] and that oxacillin-resistant isolates were associated with a severe course of the disease and poor outcome due to the limited choice of antibiotics suitable for treatment of these infections [26,27,28]. In the United States, the results of the ARMOR study indicated a prevalence of 39% methicillin-resistant *Staphylococcus aureus* (MRSA) among ocular isolates [18], while higher prevalence rates, 43% and 52.8%, were found in India and China, respectively [29,30].

Furthermore, in a study conducted by Olson et al. to determine the prevalence of methicillin resistance among staphylococcal isolates obtained from healthcare workers, a relationship between methicillin resistance and increasing age has been shown [28,31]. In our study, up to 40% of *S. aureus* isolates were found to be resistant, while higher rates of oxacillin resistance were found in CoNS isolates. 

This evidence warrants the use of drugs active against oxacillin-resistant staphylococci as empiric therapy for patients presenting with ocular infections, evaluating in each case the factors associated with an increase in resistance rates. 

In severe cases, linezolid, daptomycin and tigecycline, which show very low resistance rates, should be administered, evaluating the ability of each drug to penetrate the ocular structure involved [32,33,34,35]. Concerning the Gram-negative bacteria, fluoroquinolones have been identified as the best therapeutic choices for the treatment of ocular infections [22]; this is confirmed in this study, in which Gram-negative species showed low or no resistance to ciprofloxacin. Similar considerations apply to cefepime and the class of carbapenems.

This study did include some limitations. First, it was a cross-sectional study that reported data analysis from one single hospital centre and did not include other centres. Furthermore, some of the data without susceptibility testing were excluded. Finally, we focused on Gram-positive bacteria because approximately 80% of the isolated bacteria were CoNS and *Staphylococcus aureus*. Thus, further study is necessary to investigate the drug susceptibility of all isolates of ocular infections. 

However, in this study, a large number of ocular samples were collected, and enough species have been isolated to perform resistance analysis. 

## 4. Materials and Methods

### 4.1. Samples Collection

This cross-sectional study was conducted in the Microbiology Unit of University Hospital San Giovanni di Dio e Ruggi d’Aragona on cases recorded in the period between January 2015 and December 2019. Conjunctival samples were obtained by swabbing the lower fornix of the conjunctival sac. The eye swab was inserted into the transport media and delivered to the bacteriology laboratory, where it was processed within 3 h of collection. Out of 1364 samples, bacterial growth was obtained from 285 conjunctival swabs from patients with bacterial conjunctivitis.

### 4.2. Identification and Antimicrobial Susceptibility Testing

Conjunctival samples were inoculated on chocolate agar, blood agar, Columbia agar, MacConkey agar, Sabouraud glucose agar medium and heart–brain broth (bioMérieux, Marcy-l’Étoile, France). Only the chocolate agar plates were maintained in the presence of CO_2_. All plates were incubated at 37 °C for 18–36 h. Identification and antibiotic sensitivity tests were performed using the VITEK 2 system (bioMerieux, Marcy l’Etoile, France). Identification cards (ID-GN for Gram-Negative, ID-GP for Gram-positive, YST for yeast) and the AST-659 (for staphylococci), AST-658 (for enterococci), AST-STO3 (for *S. agalactiae*) and AST-397 (for Gram-Negative) susceptibility cards were used, according to the manufacturer’s instructions. The results of antimicrobial susceptibility tests were interpreted as “susceptible” or “resistant” according to EUCAST guidelines [36]. The quality control process encompassed the annual service and certification of the instrument by bioMérieux and the quality control of each lot of Gram-negative (GN) and Gram-positive (GP) cards using four control strains: *Enterococcus* ATCC 700,327 and *S. aureus* ATCC 29,213 for GP; and *Enterobacter* ATCC 700,323 and *Klebsiella oxytoca* ATCC 700,324 for GN.

### 4.3. Statistical Analysis

Demographic data of patients, including age, gender, isolated strain(s) and drug sensitivity results, were used for the analysis. The crude incidence and age- and sex-standardized incidences were calculated. Chi-square tests were used to verify the possible associations between the categorical variables, while the Cochran–Armitage trend test was used to verify the existence of a trend. The existence of a trend was checked only for antibiotics that showed statistically significant differences in the distribution of resistance during the years. An alpha equal to 5% was considered for both tests, so those associations that had a *p*-value < 0.05 were considered statistically significant. The IBM Statistical Package for Social Sciences Version 22.00 (SPSS Inc., Chicago, IL, USA) was used for data analysis.

### 4.4. Ethical Consideration Statement

Ethical approval by the Human Research Ethics Committee was not requested. The present study used laboratory management data collected from a database. This is a cross-sectional study, and it is not directly associated with patients.

## 5. Conclusions

In conclusion, we found a high rate of resistance to macrolides, aminoglycosides and penicillin by Gram-positive bacteria isolates. This has an important impact on the choice of empirical therapies in patients with ocular infections. Indeed, we reported a high rate of oxacillin resistance among staphylococci isolates. Our data suggest a high failure rate of beta–lactam antibiotics therapies, despite their good penetrability within ocular structures. Cotrimoxazole or tetracyclines should be considered part of the empirical treatment, and daptomycin, linezolid or tigecycline can be considered for intravenous infusion in severe cases. Finally, other studies are needed to improve the knowledge of the causative agents of ocular infections and their antimicrobial pathways for optimizing the therapeutic approach.

## Figures and Tables

**Table 1:** Conjunctival sample distribution by year.

Year	2015	2016	2017	2018	2019	2015–2019
Total samples	374	264	236	229	261	1364
Positive samples (%)	77 20.6	71 26.9	54 22.9	40 17.5	43 16.5	285 21.0

**Table 2:** Ocular infection distribution among patients according to gender and age.

Gender	% (*n*)	CI 95%
Male	54.7 (154)	[48.25–59.82]
Female	45.3 (131)	[40.18–51.75]
**Age (years)**	**% (*n*)**	**CI 95%**
0–30	25.9 (74)	[20.87–31.06]
31–60	28.1 (80)	[22.85–33.29]
61–90	45.9 (131)	[40.18–51.75]

**Table 3:** Bacteria isolated (%) from conjunctival samples of patients with ocular disease by year.

Species	2015	2016	2017	2018	2019
CoNS	55.3	60.6	47.2	43.6	35.7
*Staphylococcus aureus*	23.7	21.1	35.8	25.6	33.3
*Escherichia coli*	3.9	1.4	1.9	2.6	2.4
*Serratia marcescens*	6.6	0	3.8	2.6	0
*Pseudomonas* spp.	5.3	7.0	0	2.6	7.1
*Citrobacter* spp.	1.3	1.4	5.7	0	0
*Enterobacter* spp.	1.3	1.4	1.9	0	0
*Enterococcus faecalis*	1.3	0	3.8	2.6	7.1
*Raoultella planticola*	1.3	0	0	2.6	0
*Acinetobacter* spp.	0	1.4	0	2.6	7.1
*Klebsiella pneumoniae*	0	2.8	0	10.2	0
*Proteus mirabilis*	0	0	0	0	2.4
*Streptococcus* spp.	0	2.8	0	5.1	4.8
Total isolates (*n*)	77	71	54	40	43

**Table 4:** Resistance rates (%) of *Staphylococcus aureus* isolated from ocular samples by year.

Antibiotics	2015	2016	2017	2018	2019	*	**
Fusidic acid	0.0	13.3	15.8	0.0	0.0	0.153	0.355
Azithromycin	41.2	6.7	36.8	50.0	N.A.	0.071	0.099
Clarithromycin	41.2	6.7	36.8	50.0	N.A.	0.071	0.099
Clindamycin	44.4	13.3	31.6	30.0	50.0	0.246	0.229
Daptomycin	5.6	0.0	0.0	0.0	0.0	0.514	0.368
Erythromycin	44.4	6.7	36.8	40.0	50.0	0.113	0.157
Gentamicin	11.1	20.0	21.1	10.0	0.0	0.416	0.540
Levofloxacin	16.7	20.0	36.8	10.0	7.1	0.230	0.524
Linezolid	0.0	0.0	0.0	0.0	0.0	N.A.	N.A.
Oxacillin	33.3	13.3	26.3	40.0	14.3	0.429	0.510
Penicillin G	66.7	80.0	84.2	80.0	85.7	0.671	0.111
Rifampicin	0.0	0.0	0.0	0.0	0.0	N.A.	N.A.
Teicoplanin	0.0	0.0	0.0	0.0	0.0	0.503	0.249
Tetracycline	5.6	6.7	0.0	0.0	7.1	0.739	0.319
Tigecycline	0.0	0.0	0.0	0.0	0.0	N.A.	N.A.
Trimethoprim/Sulfam.	0.0	0.0	0.0	0.0	0.0	N.A.	N.A.
Vancomycin	0.0	0.0	0.0	0.0	0.0	N.A.	N.A.
Total isolates (*n*)	18	15	19	10	14		

* *p*-value with chi-square; ** *p*-value with Cochran–Armitage trend test; N.A., not applicable.

**Table 5:** Resistance rates (%) of coagulase-negative staphylococci isolated from ocular samples by year.

Antibiotics	2015	2016	2017	2018	2019	*	**
Fusidic acid	36.4	20.6	54.5	46.2	16.7	0.052	0.017
Azithromycin	66.7	67.6	81.8	100.0	N.S.	0.264	0.071
Clarithromycin	66.7	67.6	81.8	100.0	N.S.	0.264	0.071
Clindamycin	45.5	44.1	50.0	38.5	8.3	0.320	0.001
Daptomycin	0.0	0.0	0.0	0.0	0.0	NC	-
Erythromycin	66.7	67.6	81.8	100.0	58.3	0.087	0.002
Gentamicin	60.6	58.8	50.0	69.2	58.3	0.856	0.001
Levofloxacin	45.5	47.1	59.1	53.8	33.3	0.493	0.003
Linezolid	0.0	0.0	0.0	0.0	0.0	NC	-
Oxacillin	57.6	52.9	59.1	69.2	41.7	0.704	0.001
Rifampicin	9.1	2.9	4.5	0.0	0.0	0.548	0.027
Tetracycline	36.4	35.3	22.7	30.8	16.7	0.371	0.001
Tigecycline	0.0	0.0	0.0	0.0	0.0	NC	-
Trimethoprim/Sulfam.	0.0	5.9	0.0	0.0	0.0	0.309	0.317
Vancomycin	0.0	0.0	0.0	0.0	0.0	NC	0.017
Total isolates (*n*)	42	43	25	17	15		

* *p*-value with chi-square; ** *p*-value with Cochran–Armitage trend test; N.A., not applicable.

**Table 6:** Resistance rates of Gram-negative species isolated from ocular samples by year in %(*n*).

Antibiotics	2015	2016	2017	2018	2019	*	**
Amoxicillin/Clav. acid	71.4 (14)	80.0 (10)	57.1 (7)	28.6 (7)	0.0 (2)	0.136	0.016
Cefepime	0.0 (15)	10.0 (10)	0.0 (7)	0.0 (8)	0.0 (1)	0.080	0.001
Ceftazidime	0.0 (15)	10.0 (10)	0.0 (7)	0.0 (8)	0.0 (5)	0.528	0.479
Ciprofloxacin	0.0 (15)	9.1 (11)	0.0 (7)	0.0 (9)	0.0 (6)	0.003	0.004
Colistin	13.3 (15)	0.0 (8)	16.7 (6)	14.3 (7)	20.0 (5)	0.466	0.479
Fosfomycin	9.1 (11)	0.0 (5)	0.0 (7)	14.3 (7)	25.0 (4)	0.488	0.479
Gentamicin	6.7 (15)	36.4 (11)	0.0 (7)	0.0 (9)	0.0 (8)	0.805	0.751
Imipenem	10.0 (10)	0.0 (11)	0.0 (7)	0.0 (8)	0.0 (2)	0.047	0.006
Meropenem	6.7 (15)	0.0 (10)	0.0 (7)	0.0 (9)	0.0 (6)	0.605	0.684
Piperacillin/tazobactam	6.7 (15)	20.0 (10)	0.0 (5)	0.0 (7)	0.0 (5)	0.022	0.058
Trimethoprim/Sulfam.	21.4 (14)	41.7 (12)	0.0 (7)	0.0 (8)	0.0 (5)	0.579	0.157

* *p*-value with chi-square; ** *p*-value with Cochran–Armitage trend test; N.A., not applicable.

## Data Availability

The epidemiological data used to support the results of this study are included in the article.
